# Evolution and function of red pigmentation in land plants

**DOI:** 10.1093/aob/mcac109

**Published:** 2022-09-07

**Authors:** Kevin M Davies, Marco Landi, John W van Klink, Kathy E Schwinn, David A Brummell, Nick W Albert, David Chagné, Rubina Jibran, Samarth Kulshrestha, Yanfei Zhou, John L Bowman

**Affiliations:** The New Zealand Institute for Plant and Food Research Limited, Private Bag 11600, Palmerston North 4442, New Zealand; Department of Agriculture, Food and Environment, University of Pisa, Italy; The New Zealand Institute for Plant and Food Research Limited, Department of Chemistry, Otago University, Dunedin, New Zealand; The New Zealand Institute for Plant and Food Research Limited, Private Bag 11600, Palmerston North 4442, New Zealand; The New Zealand Institute for Plant and Food Research Limited, Private Bag 11600, Palmerston North 4442, New Zealand; The New Zealand Institute for Plant and Food Research Limited, Private Bag 11600, Palmerston North 4442, New Zealand; The New Zealand Institute for Plant and Food Research Limited, Private Bag 11600, Palmerston North 4442, New Zealand; The New Zealand Institute for Plant and Food Research Limited, Private Bag 92169, Auckland Mail Centre, Auckland 1142, New Zealand; The New Zealand Institute for Plant and Food Research Limited, Private Bag 11600, Palmerston North 4442, New Zealand; The New Zealand Institute for Plant and Food Research Limited, Private Bag 11600, Palmerston North 4442, New Zealand; School of Biological Sciences, Monash University, Melbourne, VIC, Australia

**Keywords:** Anthocyanin, antioxidant, auronidin, betalain, biosynthesis, evolution, flavonoid, photoprotection, photomodulation, stress

## Abstract

**Background:**

Land plants commonly produce red pigmentation as a response to environmental stressors, both abiotic and biotic. The type of pigment produced varies among different land plant lineages. In the majority of species they are flavonoids, a large branch of the phenylpropanoid pathway. Flavonoids that can confer red colours include 3-hydroxyanthocyanins, 3-deoxyanthocyanins, sphagnorubins and auronidins, which are the predominant red pigments in flowering plants, ferns, mosses and liverworts, respectively. However, some flowering plants have lost the capacity for anthocyanin biosynthesis and produce nitrogen-containing betalain pigments instead. Some terrestrial algal species also produce red pigmentation as an abiotic stress response, and these include both carotenoid and phenolic pigments.

**Scope:**

In this review, we examine: which environmental triggers induce red pigmentation in non-reproductive tissues; theories on the functions of stress-induced pigmentation; the evolution of the biosynthetic pathways; and structure–function aspects of different pigment types. We also compare data on stress-induced pigmentation in land plants with those for terrestrial algae, and discuss possible explanations for the lack of red pigmentation in the hornwort lineage of land plants.

**Conclusions:**

The evidence suggests that pigment biosynthetic pathways have evolved numerous times in land plants to provide compounds that have red colour to screen damaging photosynthetically active radiation but that also have secondary functions that provide specific benefits to the particular land plant lineage.

## INTRODUCTION

When and how different specialized metabolite pathways may have evolved has been a focus of much research over recent years, especially with the availability of many more non-angiosperm whole-genome sequences. A specialized metabolite stress response observed across almost all land plant taxa is the production of red pigmentation. Red pigmentation can be induced by a variety of stresses and in most tissues. In some cases, the process has been extensively characterized, such as for the reddening of leaves on deciduous trees in autumn, but there are also many open questions on red pigment biosynthesis and function. In this review, we summarize current knowledge on the evolution and function of stress-induced red pigmentation in algae and land plants, including comparing the alternative theories for cellular functions. In particular, we examine the proposal that pigment biosynthetic pathways have evolved numerous times in land plants, to provide compounds that have red colour to screen excess photosynthetically active radiation (PAR) but that also have secondary functions providing specific benefits to the particular land plant lineage.

**Fig. 1.**
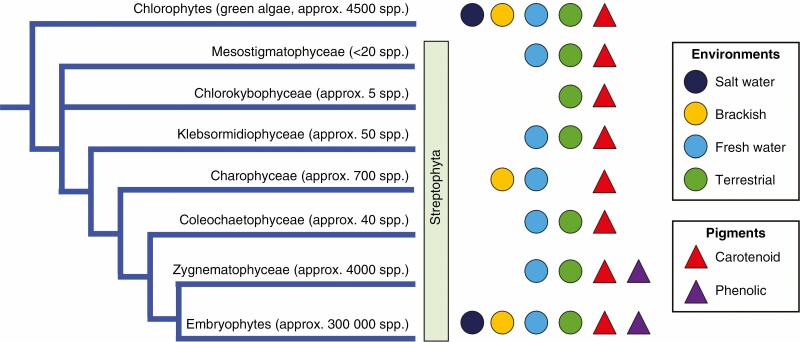
Species adapted to life on land are found in most lineages of the Viridiplantae. The currently proposed classes and relationships of the green plant lineages are shown, with estimates of the number of species within each clade. The Mesostigmatophyceae is taken as a clade containing *Mesostigma* and *Spirotaenia*. Whether species occur in the different environments is indicated. The majority of embryophyte species occur on the land, while generally low numbers of terrestrial species occur in the other clades, and often in ‘wet’ terrestrial habitats. The pigment occurrence refers to whether red- to purple-coloured pigments are produced. Many colourless simple phenolics and brownish phenolic polymers have been reported across both the Viridiplantae and more distant relatives such as the brown and red algae (e.g. phlorotannins, the oligomers of phloroglucinol common in brown algae).

The Archaeplastida group of photosynthetic eukaryotes are thought to have originated about 1500 million years ago (MYA). This gave rise to the green algae (which included the ancestors of land plants), rhodophytes (red algae) and the less species-diverse glaucophytes and Prasinodermophyta (L. Li *et al.*, 2020; Delaux and Schornack, 2021; Donoghue *et al.*, 2021; Benton *et al.*, 2022). Around 1000 MYA it is thought that the ancestors of chlorophyte algae and streptophytes arose from within the green algae lineage. The extant streptophytes are generally taken to include land plants (Embryophyta) and streptophyte algae, a group of freshwater and terrestrial algae (Fig. 1). The first land plants are thought to have arisen from a freshwater (or terrestrial) charophyte ancestor 550–470 MYA (Donoghue *et al.*, 2021).

**Fig. 2.**
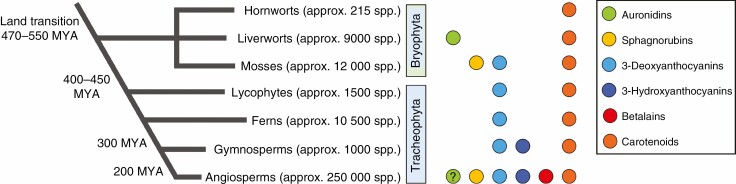
Occurrence of different red pigment types within land plants (Embryophyta). The currently proposed major classes and relationships of land plant lineages are shown, with estimates of the number of species within each clade. The occurrence of biosynthetic pathways to the betalain, carotenoid or various red phenolic pigments is indicated. The biosynthetic pathways may be of taxonomically restricted occurrence within the different groups (e.g. the restriction of betalains to the Caryophyllales), or may be reported as active only in reproductive tissues (e.g. the red carotenoid lycopene).

Early land plant evolution led to specializations for the terrestrial environment that resulted in embryophytes becoming the dominant terrestrial lineage. The extant land plants are classified into major groupings, notably the non-tracheophyte hornworts, liverworts and mosses (Bryophyta); lycophytes, ferns and fern allies (Monilophyta); gymnosperms; and flowering plants (Angiospermae) (Fig. 2). Species numbers within each of these groups vary greatly, with hornworts having the smallest estimated species diversity, at 220 (Frangedakis *et al.*, 2021), and the largest groups being mosses and flowering plants, with species estimates of >12 000 and >300 000, respectively (Benton *et al.*, 2022).

The early land plants adapted to take advantage of access to full sunlight and atmospheric CO_2_, which is available at concentrations higher than that dissolved in water and when plants first colonized land was at higher concentrations than today. However, both increased photosynthetic activity and the variable terrestrial conditions presented cellular and abiotic environment stresses different from those of the water environment, and required appropriate stress tolerance adaptations. All extant land plants (i.e. embryophytes) are thought to have evolved from a single event, resulting in the monophyletic land plant lineage. However, terrestrial colonization events have occurred independently within different algal lineages, and extant species belonging to the chlorophytes and the charophytes occupy terrestrial or semi-terrestrial habits (Fig. 1) (Holzinger and Pichrtová, 2016; Karsten *et al.*, 2016; de Vries and Archibald, 2018; Fürst-Jansen *et al.*, 2020). Some terrestrial algae are tolerant of relatively high amounts of light energy. Chlorophyte and charophyte species, plant lineages dominated by species that grow in ocean and freshwater habitats in which light penetration is reduced by the water, can tolerate >1400 μmol photons m^−2^ s^−1^ without any indication of photoinhibition (Karsten *et al.*, 2016; Hoham and Remias, 2020), and algal species have been found living in snow and ice environments where they experience much higher amounts of light energy. Based on this, it has been argued that the early algae land plant ancestors were already well adapted to a terrestrial lifestyle, and the evolution of alternative life cycles was the more influential development leading to the dominance of land plants (see Harholt *et al.*, 2016, and references therein). Another driver of the success of the land plant lineage is thought to be the development of symbiotic interactions with beneficial fungi coincident with, or soon after, the transition to the land, and it is estimated that 85 % of extant land plants form symbiotic interactions with fungi (Field and Pressel, 2018; Grosche *et al.*, 2018). Such associations are widespread in the thalloid liverworts, hornworts and vascular plants, but are generally absent in the mosses and leafy liverworts (Field and Pressel, 2018; Grosche *et al.*, 2018; Delaux and Schornack, 2021). Although algal ancestors may contain the genes that facilitate symbiosis (Delaux and Schornack, 2021), there is no evidence of fungal symbioses in charophyte green algae.

Among the abiotic stresses that are increased on land are desiccation, higher exposure to UVB radiation, and greater fluctuations in temperature, salinity and PAR. There have been recent reviews of the evolution of land plant traits as a response to these stresses (de Vries and Archibald, 2018; Fürst-Jansen *et al.*, 2020; Markham and Greenham, 2021). In this review we focus on the contributions of red pigments to tolerance of the stresses faced in a terrestrial lifestyle, including the biosynthetic diversity of red pigments and the implications of this for pigment functions and the evolutionary origin of the biosynthetic pathways.

## BIOSYNTHETIC AND STRUCTURAL DIVERSITY OF RED PIGMENTS IN PLANTS

Before discussing the diversity of red pigments in plants, it is useful to briefly cover some of the theories for the huge diversity of specialized metabolites in land plants in general, which includes >10 000 flavonoids, 12 000 alkaloids and 30 000 terpenoids (Desmet *et al.*, 2021; Kulshrestha *et al.*, 2022). A high frequency of gene duplication, with the associated opportunity for enzyme neofunctionalization to either accept new substrate combinations or generate novel products from existing substrates, is thought to have driven diversification of biosynthetic pathways (Rieseberg *et al.*, 2022). This gave both biosynthetic potential for an abundance of different possible structures within an individual plant and many lineage-specific types of specialized metabolite. However, whether each structure has evolved to be the most effective for a specific function is uncertain. It is argued that the diversity of structures is so extensive that it is untenable to suggest each has evolved a specific structure–function activity (Desmet *et al.*, 2021), and alternative theories have been proposed. The ‘Screening Hypothesis’ proposes that plants generate a diversity of structures of which only a few have biological activity. The remainder are ‘failed attempts’ at generating bioactives but are retained because the associated complexity of biosynthetic pathways increases the probability of generating new bioactives (Jones *et al.*, 1991; Firn and Jones, 2003). An addition to this, which examines how specialized metabolite biosynthetic pathways may have arisen and subsequently diversified, proposes that specialized metabolites are principally produced as components of redox chemistry, particularly for scavenging of reactive oxygen species (ROS). In this case, the presence of specific functional groups (such as accessible unsubstituted hydroxyls, particularly *ortho*-hydroxyls) is more important than the overall structure, which is ‘free’ to diversify (Hadacek *et al.*, 2010). These proposals could encompass some aspects of red pigment structural diversity. However, there is evidence for evolution favouring the production of specific complex structures for the flavonoid anthocyanin pigments, such as for generating blue flower colours (Yoshida *et al.*, 2009; Houghton *et al.*, 2021). Additionally, for *Arabidopsis thaliana* (hereafter ‘arabidopsis’), which lacks coloured flowers, >20 different anthocyanin structures have been reported (Kovinich *et al.*, 2014) as well as at least 28 different flavonoids produced in response to UVB exposure (Tohge *et al.*, 2016). The patterns of production of these are different depending on the plant growth conditions, and it has been suggested that the varied secondary modifications may promote functions suited to particular stress conditions (Kovinich *et al.*, 2014). The different challenges individual stresses create are discussed in more detail later, when we examine the relative importance of flavonoids as antioxidants that remove ROS species vs. screening out PAR or UVB radiation that contribute to generating ROS in the first place. However, there is no doubt that ROS are a central component of the damage that results from many different plant stressors.

Reactive oxygen species can be generated by various biotic and abiotic stressors, the latter of which include UVB radiation, drought, salinity, cold and excess PAR (Devireddy *et al.*, 2021; Sachdev *et al.*, 2021). Light capture can exceed CO_2_ fixation rates, and this is commonly experienced by plants in the terrestrial environment, especially when plants are simultaneously undergoing other stresses such as cold or drought that reduce the photosynthetic efficiency. The excess photon energy can generate triplet excited chlorophyll states that react with O_2_ to yield singlet oxygen (^1^O_2_·), a highly reactive radical molecule, that can cause photosystem II (PSII) inactivation and photoinhibition. In addition, under stress. ROS are mainly produced by PSI (Pospíšil, 2009), i.e. via the reduction of molecular oxygen in the Mehler reaction, a coupled process leading to the formation of ATP without NADPH (Foyer, 2018).

There are various mechanisms to cope with excess PAR, many of which are conserved across land plants (Takahashi and Badger, 2011; Pinnola, 2019; Sandmann, 2019; Bassi and Dall’Osto, 2021). These include (1) mechanisms that reduce light capture (e.g. chloroplast movement, synthesis of screening compounds and light-harvesting complex state 1 to state 2 transition) and (2) mechanisms integrated into the photosynthetic machinery aimed at mitigating the excess energy due to supernumerary photons, such as alternative electron sinks [e.g. photorespiration, cyclic electron flow around PSI and non-photochemical quenching (NPQ)], the antioxidant apparatus (mainly consisting of enzymatic and non-enzymatic antioxidants belonging to the Foyer–Halliwell–Asada cycle in chloroplasts) and the production of other antioxidant compounds (e.g. hydroxyl-rich polyphenols) to quench ROS (Agati *et al.*, 2020).

A conserved mechanism within the photosynthetic machinery for coping with excess PAR is NPQ, a multicomponent system that returns chlorophyll from the excited states back to the ground state, dissipating the excess energy as heat and thereby reducing the probability of ROS formation (for a review, see Demmig-Adams *et al.*, 2014). Carotenoids, lipophilic yellow to red coloured pigments, integrated into the photosynthetic complex are important contributors to NPQ (Pinnola, 2019; Sandmann, 2019). NPQ mechanisms and the core carotenoid biosynthetic pathway are generally conserved across plants. However, some variations among the photosynthetic proteins involved have been identified among algae, bryophytes and vascular plants, in particular the presence of light-harvesting complex stress-related proteins in algae and mosses but not vascular plants (Pinnola, 2019). In addition to quenching excited chlorophyll, carotenoids can act as antioxidants that scavenge different forms of ROS after their release from the photosystem (D’Alessandro and Havaux, 2019; Sandmann, 2019). They are one of several non-flavonoid small metabolite antioxidants, such as tocopherols, ascorbate and glutathione. In addition to non-enzymatic antioxidant compounds, the antioxidant apparatus includes a range of antioxidant enzymes, including superoxide dismutase, ascorbate peroxidase and glutathione peroxidase, forming a well-orchestrated network able to balance ROS in plant tissues (Bassi and Dall’Osto, 2021).

**Fig. 3.**
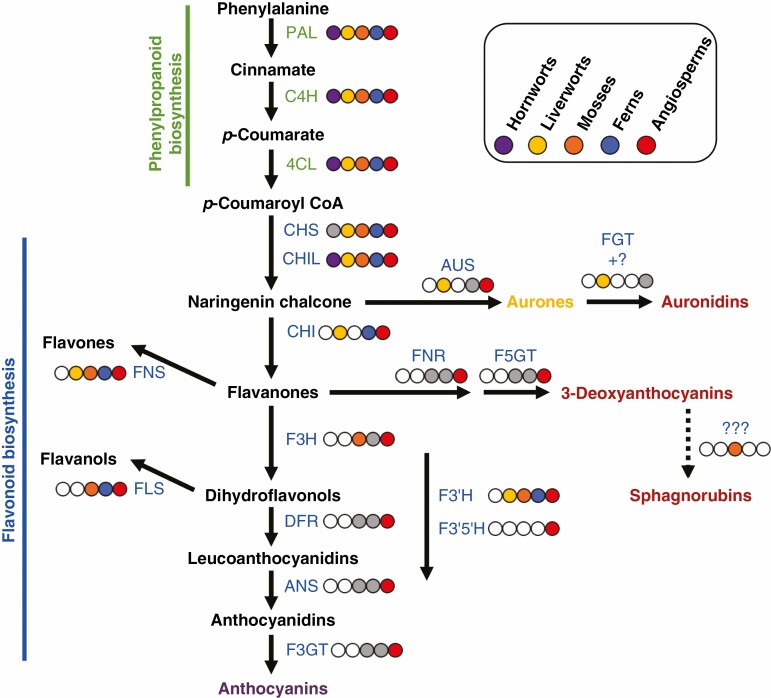
Schematic of the phenylpropanoid pathway leading to red flavonoid pigments and the occurrence of biosynthetic enzymes in different land plant lineages. A coloured circle next to an enzyme step indicates that that activity is present in the corresponding plant lineage (indicated by the colour key). A white circle with a grey outline indicates that it is not present in that lineage. A grey circle with a grey outline indicates that there is some support for the presence of the enzyme, from either sequence analysis or presence of the biosynthetic product, but that definitive evidence is lacking. With regard to proposed biosynthetic branches: the biosynthetic route to sphagnorubins is unknown but may be via 3-deoxyanthocyanidins; the biosynthetic route to auronidins is only partly characterized and, although auronidin 4-*O*-neohesperidoside can be present at relatively low amounts, auronidins principally accumulate as cell wall-bound aglycones. Enzyme abbreviations are: PAL, phenylalanine ammonia-lyase; C4H, cinnamate 4-hydroxylase; 4CL, 4-coumarate:CoA ligase; CHS, chalcone synthase; CHI, chalcone isomerase; CHIL, chalcone isomerase-like; F3H, flavanone 3-hydroxylase; DFR, dihydroflavonol 4-reductase; FNR, flavanone reductase; ANS, anthocyanidin synthase; FGTs, flavonoid *O*-glycosyltransferases; F3GT, flavonoid 3-*O*-glucosyltransferase; F5GT, flavonoid 5-*O*-glucosyltransferase; AUS, aureusidin synthase; FNS, flavone synthase; FLS, flavonol synthase; F3ʹH, flavonoid 3ʹ-hydroxylase; F3ʹ5 ʹH, flavonoid 3ʹ,5ʹ-hydroxylase.

The functions of some of the stress tolerance and antioxidant systems are disrupted by abiotic stresses such as UVB radiation that, as a downstream consequence of initial damage, alter the physiological ROS balance and promote oxidative stress (Choudhury *et al.*, 2017). This may be one driver for the evolution during land colonization of flavonoids (and the accompanying initial phenylpropanoid pathway steps) as inducible stress tolerance compounds (Yonekura-Sakakibara *et al.*, 2019; Davies *et al.*, 2020; Fürst-Jansen *et al.*, 2020; Desmet *et al.*, 2021). UVB radiation is potentially a stronger stress factor in terrestrial than aquatic environments, as UVB is absorbed by water. The Charophyceae, which are algae of high morphological organization with a totally submerged lifestyle, seem to lack UVB tolerance compounds, indicating a reliance on absorbance of UVB by the surrounding water (Holzinger and Pichrtová, 2016). UVB radiation that penetrates into cells not only generates ROS but can directly damage membranes and DNA. Without protective mechanisms, the UVB radiation amounts commonly found in direct sunlight can cause significant cellular stress, and may lead to cell or plant death. Yet, as UVB is an integral part of the sunlight that contains the PAR required for photosynthesis, avoiding exposure through shading brings other disadvantages. The majority of land plants produce phenylpropanoids such as flavonoids (Fig. 3) as a defence against UVB radiation. Flavonoids have been shown to be key for UVB tolerance in liverworts and angiosperms (Clayton *et al.*, 2018; Ferreyra *et al.*, 2021). However, because the compounds induced by UVB radiation exposure are colourless in the great majority of cases (typically flavones and flavonols), UVB tolerance mechanisms are not discussed in detail in this review. In the remainder of the article, we examine further why non-photosynthetic red pigments may be important for stress tolerance despite the presence of a range of other stress tolerance systems. We summarize the variety of red non-photosynthetic pigment structures found in plants and whether these structural variations may reflect functional variations. We conclude by discussing the different proposals for how red pigments may improve stress tolerance, and whether there may be a common mechanism across all red pigments.

### Red pigments in algae

**Fig. 4.**
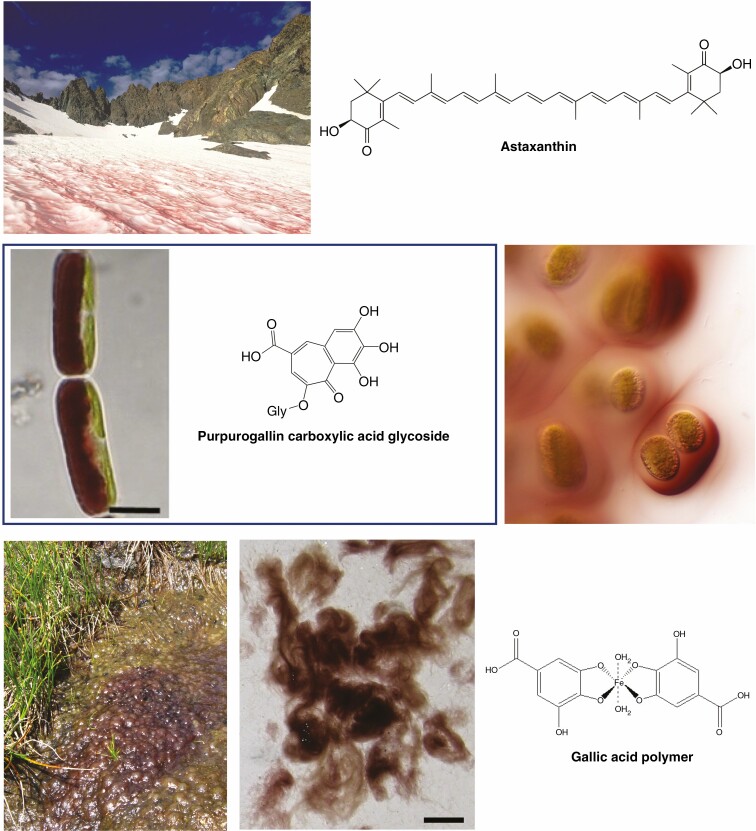
Examples of vegetative red pigmentation in terrestrial algae. The top image shows algae producing ‘blood snow’ in California, USA (USDA photograph by Paul Wade, https://commons.wikimedia.org/wiki/File:170828-FS-Inyo-PRW-001-MountRitter_(36217539154).jpg, Creative Commons Attribution 2.0 Generic licence). The middle row shows *Ancylonema nordenskioeldii* on the left and pigmented mucilage of a *Serritaenia* spp. on the right (both Zygnematophyceae). A derivative of purpurogallin has been identified as the phenolic pigment of *Mesotaenium berggrenii*, a relative of *A. nordenskioeldii*, with both these Zygnematophyceae species commonly found on alpine and Arctic glaciers and having purple-brown vacuoles containing phenolic pigments. The extracellular pigment of *Serritaenia* mucilage is also thought to be phenolic, but the structure has not been elucidated. The bottom row shows *Zygogonium erictorum* (also Zygnematophyceae), and its associated pigment thought to be a gallic acid-based polymer. Image of *A. nordenskioeldii* reprinted from Remias *et al.* (2012a). Image of *Serritaenia* courtesy of Dr Sebastian Hess (University of Cologne, Germany). Images of *Z. erictorum* reprinted from Aigner *et al.* (2013). Scale bars represent 10 µm (*A. nordenskioeldii*) and 1 cm (*Z. erictorum*).

There are diverse red pigments in algae, many of which are yet to be structurally characterized. A common algal response to abiotic stress is the production of red non-photosynthetic carotenoids such as astaxanthin and astaxanthin esters (Fig. 4) (Holzinger *et al.*, 2016; Hoham and Remias, 2020; Nakashima *et al.*, 2021). A range of algae contribute to the phenomena known as ‘blood snows’ or ‘red tides’, when the snow surface turns red from algal carotenoid production (Fig. 4), with the Chlorophyceae genera *Sanguina* and *Chlamydomonas* commonly being among the causal species (Hoham and Remias, 2020; Nakashima *et al.*, 2021). Some snow algae species produce phenolic compounds for UVB tolerance, but some are also known to produce astaxanthin in response to PAR stress, notably at the mature spore stage (Holzinger *et al.*, 2016). PAR is thought to be the key environmental trigger for astaxanthin production, usually when combined with a secondary stress such as salinity, nitrogen deficiency or fluctuations in temperature (Hoham and Remias, 2020). The excess radiant energy absorbed by the carotenoids is partly dissipated as heat. Besides cellular benefits, this may assist the algae by melting snow and releasing nutrients (Hoham and Remias, 2020). However, it also accelerates glacier wastage and contributes to climate change-associated glacial loss (Williamson *et al.*, 2020).

Zygnematophyceae species, which are one of the most recently diverged algal lineages relative to land plants, also contribute to red pigmentation of snow and ice. However, stress-induced red carotenoid pigmentation has not been recorded for the Zygnematophyceae, neither has production of mycosporine-like amino acids (MAAs) been reported for this algal group. MAAs are colourless compounds with multiple stress tolerance functions, including UVB tolerance (Carreto and Carignan, 2011). They have been extensively characterized for red algae and reported for some groups of green algae. Rather than MAAs, in response to varied abiotic stresses, water-soluble reddish pigments have been observed in the vacuoles of varied Zygnematophyceae species from alpine and glacier environments (Remias *et al.*, 2012*a*, *b*; Aigner *et al.*, 2013; Herburger *et al.*, 2016; Holzinger and Pichrtová, 2016; Williamson *et al.*, 2020). Indeed, the red pigments of terrestrial algae have been a subject of discussion in this journal since the start of the 20th century (Fritsch, 1916). The red pigments observed are thought to represent a variety of compounds, although only a few individual structures have been defined, notably a purpurogallin-derived phenolic pigment (Remias *et al.*, 2012*a*, *b*; Fig. 4). There are reports of a range of flavonoids in algal extracts (Yonekura-Sakakibara *et al.*, 2019; Jiao *et al.*, 2020). However, these have not included red flavonoid pigments, and it should be further noted that compound identification has usually been based on mass spectrometry (MS) data comparisons with databases, rather than characterization of pure compounds by structural identification methodologies such as nuclear magnetic resonance (NMR) spectroscopy. The chemistry data are supported by the proposed presence in genomes of extant algae of gene homologues for early steps of phenylpropanoid biosynthesis, although no complete gene set has been found (de Vries *et al.*, 2017; Fürst-Jansen *et al.*, 2020; Piatkowski *et al.*, 2020). Thus, some of the phenylpropanoid biosynthetic genes may have been present in the common lineage prior to the origin of land plants, while flavonoid-specific genes may have arisen only within the land plant lineage. Alternatively, these gene sequences may have arisen by convergent evolution, which also probably explains the origin of a biosynthetic pathway for a range of flavonoids in the grape endophytic fungal genus *Alternaria* (Bilska *et al.*, 2018; Lu *et al.*, 2020, 2021), as the separation of the fungi and the algae/plant ancestors is thought to have been a relatively ancient event, prior to the divergence of fungi and animals (Burki *et al.*, 2020). Convergent evolution has also given rise to the production of the red betacyanin pigments in both fungi and land plants, discussed in more detail later.

The stress-induced compound mixtures in Zygnematophyceae species may absorb in both UVB and PAR wavelengths, and pigment production can be induced by UVB radiation exposure and/or increased PAR, depending on the species (Aigner *et al.*, 2013; Holzinger and Pichrtová, 2016). Thus, assigning cellular function is difficult, and physiological data are limited. Moreover, there are no direct comparisons of red vs. non-red near-isogenic lines. However, morphs of the high-alpine species *Zygogonium ericetorum* producing purple phenolic pigments had better photosynthetic performance than green morphs (Aigner *et al.*, 2013). The red/purple morphs were dominant in the top layers of algal ‘mats’ and may help shade the green morphs underneath, which are better adapted to resist desiccation (Aigner *et al.*, 2013). Overall, the data suggest that different Zygnematophycean lineages have evolved different inducible biosynthetic pathways for red vacuolar-located pigments, comparable in many respects with the evolutionary pattern for red pigments in land plants.

Recently, the production of extracellular photoprotective pigments ranging from blackish-violet to red-brown in colour, of undefined structure, has been reported for terrestrial Zygnematophyceae species in natural environments (Busch and Hess, 2022). In a laboratory experiment, there was directional secretion of red pigment-containing mucilage towards the area of the cell exposed to UVB radiation. The pigmented mucilage was able to absorb in the UVB spectrum, although it could not be determined whether this was specifically from the red pigment or from accompanying unidentified compounds. Pigmentation was not induced in response to additional PAR generated from light-emitting diodes (LEDs); therefore, it is uncertain as to whether the ability to absorb in the visible/PAR spectrum has physiological relevance. Some cyanobacteria also produce yellowish-brown pigments in response to abiotic stresses such as UVB radiation, excess PAR, desiccation and salinity, most notably the indole-alkaloid scytonemin that accumulates in extracellular polysaccharides (Storme *et al.*, 2015; Ferreira and Garcia-Pichel, 2016). Scytonemin is able to absorb both PAR and UVB radiation, and has been widely studied in relation to UVB tolerance. As with the extracellular Zygnematophyceae pigment, these can also be produced in a directional manner towards the light stress, presenting a further example of convergent evolution for photoprotective pigmentation (Storme *et al.*, 2015; Busch and Hess, 2022).

### Flavonoid red pigments

**Fig. 5.**
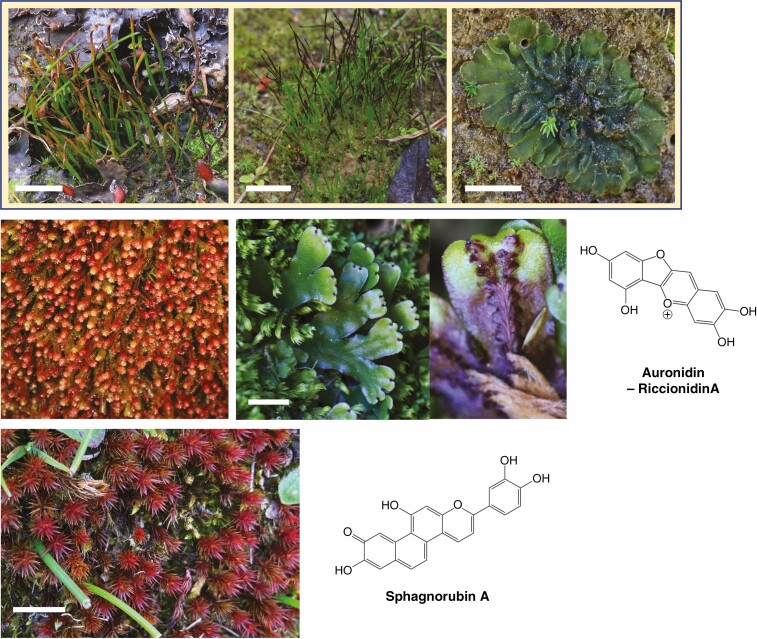
Examples of vegetative red pigmentation in non-tracheophyte plants. Example species are shown for hornworts (top row: from left to right; *Phaeoceros carolinianus*, *Anthoceros laminifer* and *Phaeomegaceros hirticalyx*), liverworts (middle row: *Isotachis* spp. on left and *Marchantia foliacea* on right) and mosses (bottom row: *Polytrichum juniperinum*/Juniper haircap moss) alongside structures of red pigments that are produced. The specific plant species shown may produce variations of the example core structure. No red pigment has been reported for hornworts. Scale bars represent 1 cm. All photographs by the authors.

**Fig. 6.**
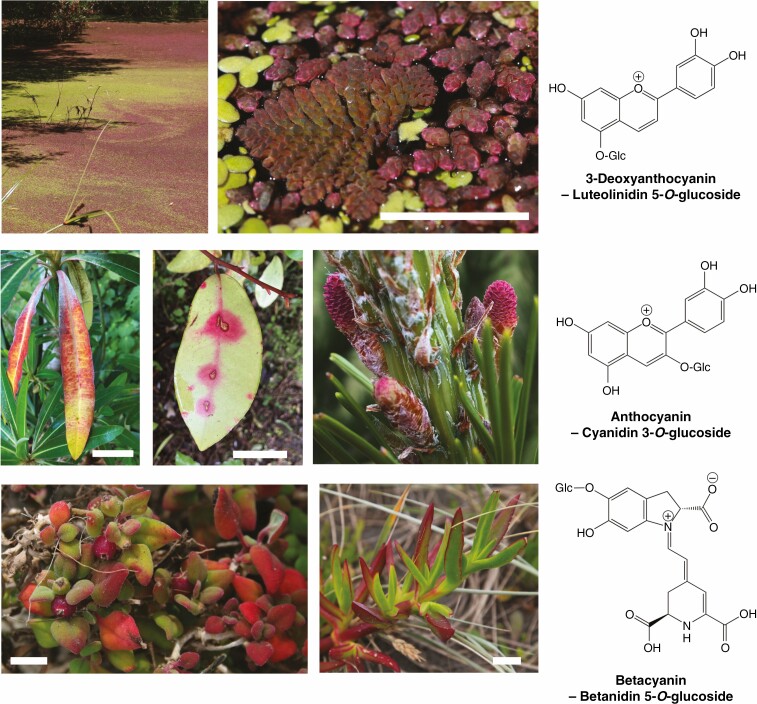
Examples of vegetative red pigmentation in vascular plants. Top row: the fern *Azolla* covering a pond in full sunlight and showing 3-deoxyanthocyanin production. The close-up image shows *Azolla pinnata* – triangular, regularly branched plant to the left of the image –and *Azolla rubra*, mixed with the green angiosperm *Lemna minor* (duckweed). Middle row: the angiosperms *Euphorbia mellifera* (Canary spurge/honey, left) and *Pseudowintera colorata* (centre), with pathogen/pest-induced anthocyanin production. Also shown are the red reproductive cones of the gymnosperm *Pinus radiata* (right). Bottom row: *Tetragonia trigyna* (left) and *Carpobrotus* spp. (right, ice plant, probably *C. edulis*, although hybrids can form with *C. chilensis*, which was co-occurring). The specific plant species shown may produce variations of the example core structure (such as glycosylation or other modifications). Scale bars represent 2.5 cm except for *Azolla*, where it is 1 cm. All photographs by the authors except *P. radiata* (Lorelle Phillips, Scion, New Zealand).

**Fig. 7.**
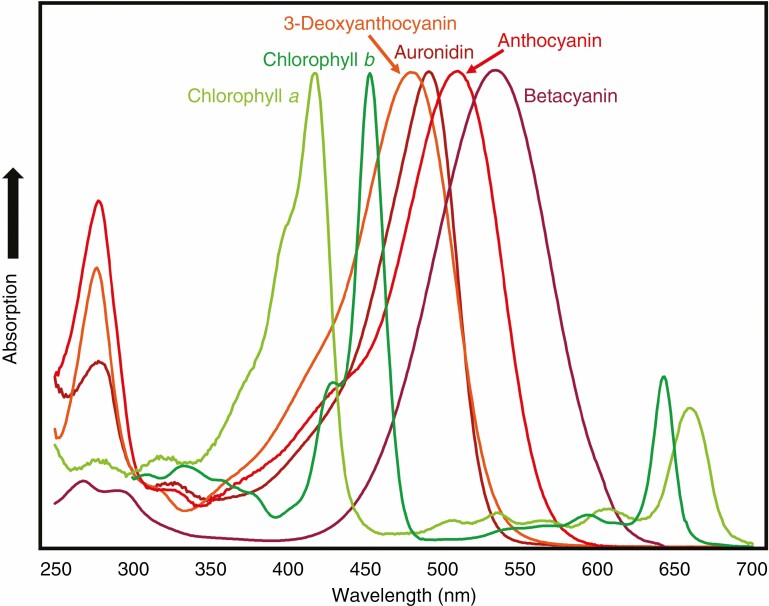
Comparative absorption spectra of chlorophyll *a*, chlorophyll *b* and different red pigments of land plants. Example spectra are shown for an auronidin (riccionidin A), a 3-deoxyanthocyanin (luteolinidin), a 3-hydroxyanthocyanin (kuromanin; cyanidin-*O*-glycoside) and a betalain (betanin; betanidin-5-*O*-glucoside).

**Fig. 8.**
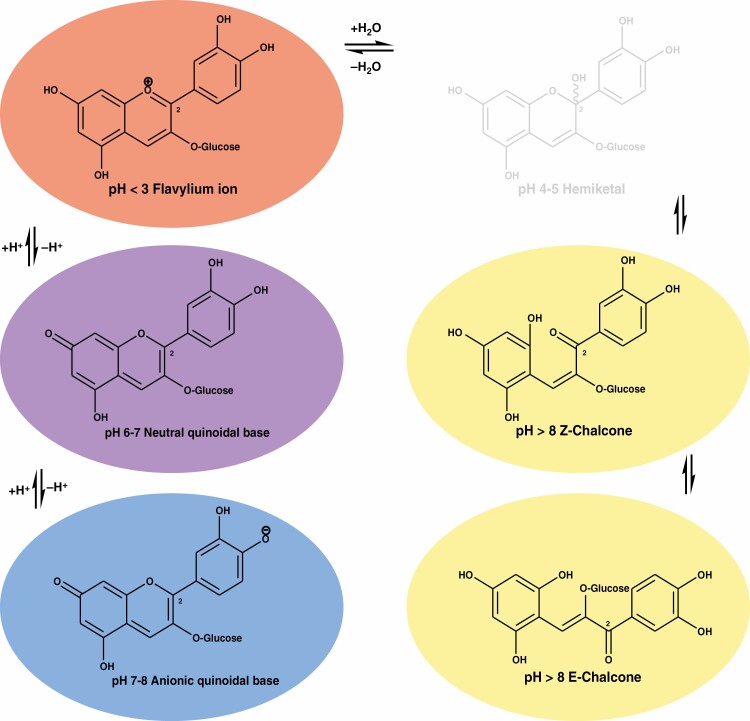
Structural modification and associated colours of the anthocyanin cyanidin-3-*O*-glucoside as a function of pH and hydration. Colour change from strong acid to base is shown, together with the causal shifts in chemical form.

The main red pigments of most land plants are anthocyanins (Figs 5–8). Anthocyanins are formed in a branch of the flavonoid pathway (Fig. 3), which has been reported from all land plant groups except hornworts. Flavonoids have a C6–C3–C6 core with two aromatic rings (named A- and B-rings) linked by a heterocyclic ring (C). The flavonoids are sub-divided into various classes based on the pattern of oxygenation of the core structure, with additional hydroxylation and/or methylation contributing to different core structures within each class. Classes commonly associated with tolerance to abiotic stress include the typically colourless flavones and flavonols, and the pigmented anthocyanins, 3-deoxyanthocyanins, auronidins and sphagnorubins. When considering land plants as a whole, anthocyanins are produced in a plethora of structures, in almost every type of plant organ, and in response to many different developmental and environmental signals. The variety of anthocyanin production in plants has given rise to a corresponding variety of theories on function, which include photomodulation, thermoregulation, anti-pathogen activity, defence against herbivores, sinks for excess carbon, osmoregulation, metal chelation and communication to pollen and seed dispersal agents.

The vast majority of anthocyanins are based around six core structures – anthocyanidin aglycones – which differ in the number and position of hydroxyl groups and whether those groups are methylated. The two major sub-groups are the ‘typical’ anthocyanins (3-hydroxyanthocyanins) and the 3-deoxyanthocyanins (Fig. 6). In this review, and commonly in the literature, unless otherwise specified, the term ‘anthocyanin’ refers to 3-hydroxyanthocyanins. A key feature of these is the number of hydroxyl groups on the B-ring, with 3ʹ-, 3ʹ,4ʹ- or 3ʹ,4ʹ,5ʹ-hydroxylation distinguishing pelargonidin-, cyanidin- and delphinidin-based anthocyanins, respectively. Extensive secondary modifications of the core anthocyanidin structure are known, principally glycosylation with a range of hexose moieties and secondary aromatic and aliphatic acylation of the carbohydrates. To date, around 800 different anthocyanin structures have been reported (Andersen and Jordheim, 2006; Berland and Andersen, 2021). Both the anthocyanidin aglycone structure and the patterns of secondary modifications influence the resultant colour of the plant tissue. Some of the structural modifications provide anthocyanins that can generate the colours preferred by specific pollinators, such as the highly modified delphinidin anthocyanins that are common to most blue flowers (Andersen and Jordheim, 2006; Yoshida *et al.*, 2009). Whether the structural variations are important for stress tolerance properties is uncertain. The great majority of anthocyanins reported for vegetative tissues of vascular plants are cyanidin based, and confer red colour to the tissue. Similarly, in ferns, the 3-deoxyanthocyanins characterized in vegetative tissues are principally the 3ʹ,4ʹ-hydroxylated luteolinidin (Cohen *et al.*, 2002*b*; Andersen and Jordheim, 2006).

The 3-deoxyanthocyanins occur in only some angiosperm taxa, where they can provide bright orange flower colours and/or plant defence compounds (Winefield *et al.*, 2005; Liu *et al.*, 2010; Kariyat *et al.*, 2019; Xiong *et al.*, 2019). Reports of 3-deoxyanthocyanins from ferns or angiosperms are nearly all of simple glycosides, although there are a few reports of acylated compounds (Andersen and Jordheim, 2006). Bright red pigmentation from 3-deoxyanthocyanins is characteristic of the water fern *Azolla* (Cohen *et al.*, 2002*a*, *b*). In addition to having a physiological *in planta* function, the pigments may also be part of the signalling pathway to the symbiotic cyanobacterium *Nostoc* (Cohen *et al.*, 2002*b*). The 3-deoxyanthocyanins have also been reported to be present in some mosses (Andersen and Jordheim, 2006). The importance of these types of 3-deoxyanthocyanins to moss pigmentation is unclear, as mosses also produce another group of red pigments – the sphagnorubins, which are flavonoid based. Sphagnorubins have two additional aromatic rings connected to the A-ring (Fig. 5). As sphagnorubins contain a 3-deoxyanthocyanin component to their structure, it is possible that they are derived from 3-deoxyanthocyanins through the action of additional biosynthetic steps absent in other land plants. However, this is an assumption until the genetics of the sphagnorubin biosynthetic pathway are revealed. Phylogenetic analysis has suggested that mosses lack some of the biosynthetic genes required in angiosperms for 3-deoxyanthocyanin production (Piatkowski *et al.*, 2020). Until recently, it was assumed the red flavonoid pigments of liverworts, as typified by riccionidin A (Fig. 5) were anthocyanins. However, Berland *et al.* (2019) demonstrated that the red pigments of liverworts are biosynthesized from a previously unreported branch of the flavonoid pathway. As this pathway proceeds via aurones, they named the pigments auronidins.

Sphagnorubins have been reported only as cell wall-bound compounds *in planta*. They are assumed to accumulate as aglycones, without secondary modifications. In contrast to the common anthocyanins, sphagnorubins are not soluble in water. Similarly the great majority of reports of auronidins also describe them as cell wall-bound compounds, presumed to be aglycones. A neohesperidose of auronidin (a glucose attached to a rhamnose that is attached to the auronidin) has been identified, although this was from a transgenic line with greatly increased auronidin biosynthesis (Berland *et al.*, 2019).

Anthocyanins are by far the most common red pigments produced in the majority of gymnosperms and angiosperms. Common anthocyanins have been extensively characterized for the shifts in chemical form that occur, both *in vitro* and *in planta*, in response to the local hydration environment of the molecule and the effect of pH (Andersen and Jordheim, 2006; Alejo-Armijo *et al.*, 2020). They convert between flavylium cation, quinoidal base, hemiketal, and *cis*- and *trans*-chalcone forms that differ in the perceived colour and pigment stability. Under the common *in vitro* conditions studied, the flavylium cation is red, the quinoidal base is purple and the mono-cationic quinoidal base is blue (Fig. 8). More recently, the *in vitro* chemistry of anthocyanins, 3-deoxyanthocyanins and auronidins (riccionidin A aglycone, classified as a furanoflavylium cation) have been compared for insights on what advantages each pigment could offer *in planta* (Alejo-Armijo *et al.*, 2020; Pina *et al.*, 2021). All three pigment types can undergo a similar sequence of chemical transformations, involving proton transfer, hydration, tautomerization and *cis*–*trans* isomerization. However, there are significant differences in kinetics and thermodynamics, as well as the resultant colours. Notably, the rates for interconversion between the auronidin species are dramatically slower. Sphagnorubin C has recently been found to behave *in vitro* as a two-component colour system between the reddish flavylium cationic and the yellow *trans*-chalcone forms, with the hemiketal and *cis*-chalcone forms appearing only transiently under equilibrium conditions (Berland and Andersen, 2021). The lack of the hydroxyl, and associated glycosyl, groups at the C-3 position in 3-deoxyanthocyanidins and sphagnorubins provides significantly increased stability against hydrophilic attack compared with 3-hydroxyanthocyanins (Berland and Andersen, 2021; Pina *et al.*, 2021).

There are few studies on the vegetative production of anthocyanins in response to stress in gymnosperms, which could reflect its being a less common phenomenon. Anthocyanins are common in gymnosperm reproductive tissues, including the fleshy arils of species such as yew (*Taxus baccata*), fleshy false arils in species such as several of the Podocarpaceae of New Zealand, and seed cones of many conifer species (Rudall, 2020). Anthocyanins are generally produced during the earlier stages of cone development (Fig. 6). Amounts have been found to increase under environmental conditions that can generate more PAR-related stress, such as increasing altitude or periods of bright days and cold nights (Rudall, 2020). Theories for the anthocyanin function in cones are similar to those for anthocyanins in vegetative tissues – photomodulation, thermoregulation or defence against herbivores.

### Evolution of the biosynthetic pathway for red flavonoid pigments

The sequencing of genomes from bryophytes and lycophytes, and the identification of the auronidin branch, has contributed to clearer proposals for how the flavonoid pathway evolved in land plants. The core phenylpropanoid pathway of PHENYLALANINE AMMONIA-LYASE (PAL), CINNAMATE 4-HYDROXYLASE (C4H) and 4-COUMAROYL-CoA LIGASE (4CL) that converts phenylalanine to the flavonoid precursor *p*-coumaroyl-CoA is conserved across land plants. The first committed flavonoid biosynthetic step is carried out by CHALCONE SYNTHASE (CHS), a POLYKETIDE SYNTHASE (PKS) Type III enzyme (Kim *et al.*, 2013; Morita *et al.*, 2019; Yonekura-Sakakibara *et al.*, 2019; Naake *et al.*, 2021). *PKS* genes with high sequence similarity to *CHS* are present in all land plant genomes examined to date (Yonekura-Sakakibara *et al.*, 2019; Piatkowski *et al.*, 2020; Davies *et al.*, 2021; Naake *et al.*, 2021). It is known for all major land plant groups, with the exception of hornworts, that at least some of these *PKS* genes encode CHS. The *Anthoceros* hornwort genomes (F.W. Li *et al.*, 2020; Zhang *et al.*, 2020) do contain *PKS* sequences (Davies *et al.*, 2021), but they have yet to be examined by either assay of the encoded enzymatic activities or loss-of-function mutations. Analyses of *PKS* sequences in liverworts (Yu *et al.*, 2018) and mosses (Kim *et al.*, 2013) have identified authentic *CHS* genes but also other genes encoding different PKS activities. CHS activity cannot be assigned with certainty by sequence similarity alone, as minor alterations to the active site can switch the catalytic function. There are >20 different PKS enzymes identified to date in plants, and a few amino acid substitutions have been demonstrated to change activities from CHS to activities such as ACRIDONE SYNTHASE or STILBENE SYNTHASE (Morita *et al.*, 2019; Naake *et al.*, 2021). Thus, PKS sequences frequently form phylogenetic clades on a species, rather than a functional, basis (Kim *et al.*, 2013; Davies *et al.*, 2021).

Auronidins are red flavonoid pigments synthesized in a pathway branch that proceeds from chalcones via the yellow aurone intermediates (Fig. 2). It is thought that all other flavonoid pigments proceed via the conversion of chalcones to flavanones. This can occur spontaneously *in vitro*, but *in planta* the enzyme CHALCONE ISOMERASE (CHI) is generally required for significant flavanone formation. This is evidenced by the marked reduction or loss of flavanone-derived flavonoids in *chi* mutants in bryophyte (Clayton *et al.*, 2018) or flowering plant species (Jiang *et al.*, 2015). However, there are important open questions regarding CHI and the presence/absence of *CHI* genes in the bryophytes. As mentioned, liverworts have *CHI* genes and the loss-of-function mutants in *Marchantia polymorpha* lose production of flavanone-derived flavonoids. The published hornwort genome sequences (*Anthoceros* spp.) lack *CHI* genes (Davies *et al.* 2020, and the authors’ unpublished analyses). Thus, this is a possible basis for the lack of flavonoids in hornworts. However, moss genome sequences also lack *CHI* genes (Cheng *et al.*, 2018), but mosses produce abundant flavonoids. A proposal we consider worthy of investigation is that mosses have evolved an alternative mechanism for conversion of chalcones to flavanones but hornworts have not.

Why hornworts and mosses lack *CHI* genes is not clear. Both contain the related *CHALCONE ISOMERASE-LIKE* (*CHIL*) genes, which share with CHI a common evolutionary origin from genes encoding fatty acid-binding proteins. Additionally, the intron/exon structure of *CHIL*, including the intron that interrupts the starting ATG, is conserved between all the bryophytes and arabidopsis (the authors’ unpublished analyses), as is the intron/exon structure of *CHI* between *M. polymorpha* and arabidopsis (Bowman *et al.*, 2017). If the proposed monophyletic grouping of bryophytes is accepted, this suggests that both *CHI* and *CHIL* genes were present in the last common ancestor of extant land plants but *CHI* was lost during the evolution of mosses and hornworts. CHIL is a non-catalytic protein that can promote the production of various groups of phenylpropanoids, including flavonoids. In species examined to date, it can bind to CHS to direct the potentially promiscuous CHS activity towards formation of chalcones (Waki *et al.*, 2020; Dastmalchi, 2021). There are loss-of-function *chil* mutants in both angiosperms (Morita *et al.*, 2014) and liverworts (Clayton *et al.*, 2018; Berland *et al.*, 2019) that have greatly reduced flavonoid production. CHIL may also interact with other enzymes. In hops, CHIL promotes activity of both CHS and an aromatic prenyltransferase (Ban *et al.*, 2018). In arabidopsis, it is proposed to promote flavonoid biosynthesis through interaction with CHS and CHI (Jiang *et al.*, 2015). CHIL is unlikely to be the basis of flavonoid formation in the absence of CHI, as moss *CHIL* genes assayed *in vitro* do not have CHI activity (Cheng *et al.*, 2018).

Flavanones are the starting point for pathway branches to 3-deoxyanthocyanins, 3-hydroxyanthocyanins, flavones and flavonols. Flavones are produced directly from flavanones by a range of enzymes (Yonekura-Sakakibara *et al.*, 2019; Davies *et al.*, 2020; D.D. Li *et al.*, 2020). In angiosperms, 3-deoxyanthocyanidins are formed by FLAVANONE 4-REDUCTASE (FNR) converting flavanones to flavan-4-ols, which are substrates for ANTHOCYANIDIN SYNTHASE (ANS) and then UDP-GLYCOSIDE:FLAVONOID 5-*O*-GLYCOSYLTRANSFERASE (UF5GT) (Winefield *et al.*, 2005; Shih *et al.*, 2006; Ibraheem *et al.*, 2015). Compared with 3-deoxyanthocyanins and flavones, the biosynthetic sequence to 3-hydroxyanthocyanins and flavonols includes an additional enzyme step following CHI: FLAVANONE 3-HYDROXYLASE (F3H), which makes dihydroflavonols (Fig. 3). The subsequent steps for 3-hydroxyanthocyanins are analogous to those for 3-deoxyanthocyanins: DIHYDROFLAVONOL 4-REDUCTASE (DFR), ANS and UDP-GLYCOSIDE:FLAVONOID 3-*O*-GLYCOSYLTRANSFERASE (UF3GT). Liverworts lack *F3H* genes, and do not produce either 3-hydroxyanthocyanins or flavonols (D.D. Li *et al.*, 2020). *F3H* genes are present in moss genomes, and they produce flavonols. Despite having F3H, mosses do not produce 3-hydroxyanthocyanins. The reasons for this are unknown. Nor is the biosynthetic pathway to the red sphagnorubin pigments of mosses known.

Mosses, ferns and lycophytes produce 3-deoxyanthocyanins, but liverworts do not. However, neither the basis for their absence in liverworts nor the genes involved in their production in mosses, lycophytes or ferns has been established. The lack of an *FNR* gene has been suggested to be the basis for the absence of 3-deoxyanthocyanins in some plant groups (Piatkowski *et al.*, 2020). Yet in the angiosperm species examined for 3-deoxyanthocyanin biosynthetic pathways to date, FNR and DFR are a single dual-functional enzyme. The FNR/DFR of *Sinningia cardinalis*, which has 3-deoxyanthocyanin-pigmented flowers, has similar activity to flavanones or dihydroflavonols (Winefield *et al.*, 2005), and loss of function of the *A1* FNR/DFR gene in maize causes loss of production of both types of anthocyanin (Halbwirth *et al.*, 2003; Morohashi *et al.*, 2012). Dual-function FNR/DFR genes have also been reported for *Sorghum* (Liu *et al.*, 2010), although a gene encoding a protein with only FNR activity has been found, and mutations in that gene caused loss of 3-deoxyanthocyanin production (Kawahigashi *et al.*, 2016).

All land plant groups (except hornworts) produce some flavonoids with *ortho*-hydroxylation of the B-ring (3ʹ4ʹ-hydroxylation), and those flavonoids associated with UVB tolerance or vegetative red pigmentation commonly have this hydroxylation pattern. In all characterized cases, this is catalysed by FLAVONOID 3ʹ-HYDROXYLASE (F3ʹH, the cytochrome P450 clade CYP75B), which accepts varied 3ʹ-hydroylated substrates. Some angiosperms also have the related enzyme FLAVONOID 3ʹ,5ʹ-HYDROXYLASE (F3ʹ5ʹH, CYP75A), and so produce flavonoids with 3ʹ4ʹ5ʹ-hydroxylation of the B-ring, most notably delphinidin-derived anthocyanins. Piatkowski *et al.* (2020) identified a *F3ʹH*/*F3ʹ5ʹH* orthogroup basal to land plants and charophyte green algae but absent from seedless plants. They also discuss the challenges of using sequence-based approaches for this gene clade. Indeed, despite several phylogenetics-based reports of *F3ʹ5ʹH*-like sequences in seedless plants, the only *F3ʹ5ʹH* genes verified by recombinant protein assays to date are from angiosperms. Zhang *et al.* (2020) found that the *CYP71* subfamily was highly expanded in *Anthoceros angustus*, consisting of 56 gene models, including 32 genes encoding F3ʹH or F3ʹ5ʹH. However, the accompanying phylogenetic analysis did not place any sequences in the CYP75 clade, and our analysis also indicates the absence of strong *F3ʹH* or *F3ʹ5ʹH* candidates in hornworts. The analysis of de Vries *et al.* (2021) placed 72 *Anthoceros CYP* sequences within a larger clade containing, for example, arabidopsis sequences of *CYP71*, *CYP75* and *CYP76* (and a sub-clade of characterized bryophyte *CYP98A CINNAMATE 3-HYDROXYLASE* sequences). If bryophytes do lack *F3ʹH* genes, they may have evolved alternative hydroxylation activities, perhaps acting on the phenylpropanoid precursors before they become substrates for CHS. The majority of flavonoids are modified by the addition of one or more hexose groups (glycosylation), and may also have other secondary modifications through acylation, methylation, prenylation and sulfation. Patterns of flavonoid secondary modification vary among and within the major plant groups (Alseekh *et al.*, 2020), but the evolutionary basis for this variation is generally unresolved.

The diversity of flavonoids across land plants makes it difficult to suggest what the flavonoid biosynthetic gene set may have been in the last common ancestor of extant land plants. Phylogenetics-based approaches to identify orthologue groups can help predict the shared genes among land plants that may represent the minimal set present in the last common ancestor, as comprehensively presented recently for flavonoid biosynthesis by Piatkowski *et al.* (2020). However, given the presence of genes within the flavonoid pathway with similar sequences but differing functions, functional characterization of the candidates revealed by phylogenetics is then often necessary. For example, phylogenetics identified *F3H* in liverworts but not mosses (Piatkowski *et al.*, 2020), but assays of recombinant proteins suggest the reciprocal situation (D.D. Li *et al.*, 2020). With regard to biosynthetic gene occurrence for red flavonoid pigments: hornworts possibly lack any flavonoids; non-tracheophyte plants lack 3-hydroxyanthocyanins; 3-deoxyanthocyanins have a sporadic distribution; and auronidins and sphagnorubins may be limited to liverworts and mosses, respectively. This pattern probably represents a mix of gains and losses of pathway branches since the last common ancestor. There are relatively few flavonoid biosynthesis components confirmed as common across all land plants, these being CHIL and PKS and the core phenylpropanoid pathway of PAL, C4H and 4CL. CHS and UDP-glycosyltransferase activities have been confirmed for all groups except hornworts to date. If it is assumed (based on the monophyly of bryophytes) that hornworts have lost the ability to make flavonoids, then it is probable that the last common ancestor had the genes required for formation of flavones, which could have been part of UVB tolerance. It is more difficult to draw conclusions for red pigmentation, except that the production of 3-hydroxyanthocyanins is a later evolutionary innovation (as is betalain biosynthesis). The evolutionary driver to have stress-related red pigmentation seems very strong, given its occurrence across many algae and nearly all land plants. As biosynthetic routes to red are present in almost all land plants, the last common ancestor of land plants may have had red pigmentation. However, there is insufficient information to propose which red pigment biosynthetic pathway may have been present. If the 3-deoxyanthocyanin biosynthetic genes of mosses and lycophytes are found to have a common genetic origin with those of angiosperms, this would support an early evolutionary origin of that pigment group. However, recombinant assays using the *F3H* genes of *Psychomitrella patens* (a moss) and *Selaginella moellendorffii* (a lycophyte) showed that they encode enzymes with dual FNSI/F3H activities (D.D. Li *et al.*, 2020), suggesting that the seed plant *F3H* and non-seed plant *FNSI/F3H* genes are the result of independent evolutionary events from a common ancestral 2-oxoglutarate-dependent dioxygenase gene with a different function.

### Blue, yellow and orange flavonoid pigments in relation to stress responses

Anthocyanins can confer blue pigmentation, commonly based on delphinidin production (Yoshida *et al.*, 2009). There are also bright yellow flavonoid pigments – most notably aurones. However, neither delphinidin nor aurone production is a commonly characterized response to stress. The anthocyanins produced in response to stress are usually cyanidin based, rather than pelargonidin-, peonidin- (methylated cyanidin) or delphinidin-derived anthocyanins. The evidence suggests that biosynthesis of 3-hydroxyanthocyanin alternatives to cyanidin may have arisen only for plant communication with the beneficial biotic environment, specifically for pollination and seed dispersal. The simple delphinidin-based anthocyanins do not generate blue colours at physiological pH, and a great many adaptations that generate blue anthocyanin-based pigmentation have been characterized. These include anthocyanin secondary modifications and co-pigmentation changes that alter the local environment of the pigment, and genes that directly increase the vacuolar pH (Yoshida *et al.*, 2009; Houghton *et al.*, 2021). However, these are all associated with formation of blue flower or fruit colours rather than stress responses.

The auronidin aglycone can form colours from yellow through to purple *in vitro*, but only at about pH 1.0 and pH 12.0, respectively (Alejo-Armijo *et al.*, 2020), and have only been reported as red compounds *in planta*. No blue flower colours from 3-deoxyanthocyanins have been reported from nature and, even *in vitro* at more basic pH values, luteolinidin does not generate blue colours (Alejo-Armijo *et al.*, 2020). The retention of 3-deoxyanthocyanin biosynthesis in some angiosperms despite the evolution of the 3-hydroxyanthocyanin branch suggests some distinct functions. In some species, they confer bright orange/scarlet colours that favour bird-mediated pollination (Winefield *et al.*, 2005; Xiong *et al.*, 2019). Whether they are also the anthocyanins produced in response to abiotic stress in these species has not been examined in detail (Xiong *et al.*, 2019). However, various 3-deoxyanthocyanin compounds are produced in vegetative tissues as phytoalexins, notably in the Andropogoneae grasses such as sorghum and maize (Liu *et al.*, 2010; Kariyat *et al.*, 2019; Xiong *et al.*, 2019). They also contribute to feeding deterrence against snails and tadpoles in the fern *Azolla*, as well as promoting symbiosis with the nitrogen-fixing cyanobacterium *Nostoc* (Cohen *et al.*, 2002*a*, *b*).

The yellow flavonoids – the chalcones, aurones and some substituted flavonols of infrequent occurrence (Boucherle *et al.*, 2017) – have not been reported as stress-related pigments. Indeed, they are generally not reported as accumulating in significant amounts in non-reproductive tissues. The aurones are the most taxonomically widespread and brightest yellow of the flavonoid pigments. They can provide nectar guides to pollinators, but the reasons for production in non-floral structures such as liverwort sporophytes (Markham and Porter, 1978) are unknown. There is a similar knowledge gap for the function of yellow pigments in the next group of compounds we discuss – the betalains.

### Betalains

Betalain pigments have limited distribution, with convergent evolution giving rise to their presence in only a few fungal and plant lineages (G. Li *et al.*, 2019; Timoneda *et al.*, 2019; Tossi *et al.*, 2021). They are water-soluble nitrogen-containing compounds and, like anthocyanins, are stored in the vacuole. In plants, they have evolved to replace anthocyanins in several families within the core Caryophyllales (Brockington *et al.*, 2011; Timoneda *et al.*, 2019). The Caryophyllales taxa contain proportionally more species adapted to living in extreme (e.g. arid or saline) environments than reported for angiosperms overall, and these include many betalainic species (G. Li *et al.*, 2019). The Caryophyllales are also known for the high frequency of occurrence of Crassulacean acid metabolism (CAM), an adaptation to periodic water supply, and C_4_ photosynthesis (Christin *et al.*, 2015), as well as halophytism/Na^+^ hyperaccumulation (Flowers and Colmer, 2008; Moray *et al.*, 2015; White *et al.*, 2017). Indeed, salt tolerance may have evolved early in some betalain lineages and then been retained during species radiation (Moray *et al.*, 2015). It is possible that betalain biosynthesis also evolved in response to the challenging environments that these Caryophyllales species occupy. There are several betalain-producing species that are generally familiar as food or ornamental crops, including beet/chard (*Beta vulgaris*), amaranth (*Amaranthus caudatus*) and quinoa (*Chenopodium quinoa*) of the Amaranthaceae; prickly pear (*Opuntia* spp) and dragon fruit (*Hylocereus/Selenicereus* spp.) of the Cactaceae; moss rose (*Portulaca grandiflora*, Portulacaceae); iceplants (various Aizoaceae spp.); and bougainvillea (*Bougainvillea glabra*) and four o’clock flower (*Mirabilis jalapa*) of the Nyctaginaceae.

**Fig. 9.**
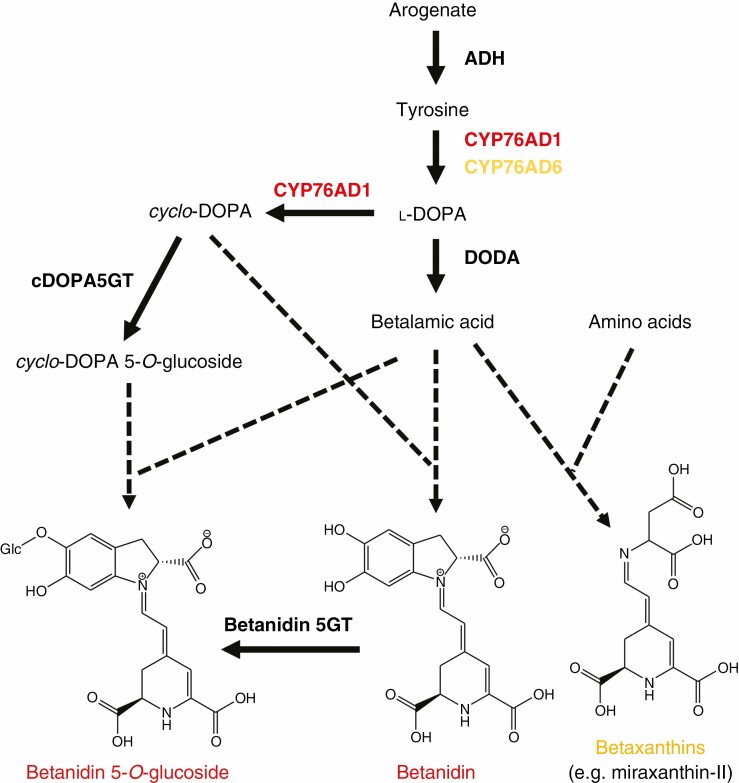
Schematic of the biosynthetic pathway to betacyanins and betaxanthins. Enzymes shown are the cytochrome P450 enzymes CYP76AD1 and CYP76AD6, DOPA 4,5-dioxygenase (DODA), *cyclo*-DOPA 5-*O*-glucosyltransferase (cDOPA5GT) and betanidin 5-*O*-glucosyltransferase (Betanidin 5GT). The condensation reactions to form betacyanin and betaxanthin pigments occur spontaneously (shown as dashed lines).

The central chromophore of betalains is the pale yellow compound betalamic acid (BA), which is derived from tyrosine (Fig. 9). Although BA accumulates in some betalainic plants, it commonly is converted to red-violet betacyanin pigments through its condensation with *cyclo*-dihydroxyphenylalanine (*cyclo*-DOPA) (this may be BA aglycone or glucoside, or less commonly a decarboxylated form), and to yellow betaxanthin pigments when condensed with amino acids or amines. Betaxanthins and betacyanins have absorption maxima in the ranges 532–550 nm and 457–485 nm, respectively. The condensation reactions appear to be spontaneous rather than enzyme catalysed (Schliemann *et al.*, 1999). Like anthocyanins, the core betacyanin structure is commonly modified by glycosylation and acylation. While the function of some secondary modifications is understood for anthocyanins, data for betacyanins are lacking. Unlike anthocyanins, glycosylation is not required for stable cellular accumulation of betalains, as in some Caryophyllales species and in some transgenics of anthocyanic species engineered to produce betalains the non-acylated aglycone betanidin and/or related isomers accumulate (Strack *et al.*, 2003; Chung *et al.*, 2015; Polturak *et al.*, 2016, 2017; Grützner *et al.*, 2021; Zhou *et al.*, 2021). However, secondary modifications can alter the colour properties of betacyanin and may increase stability at varied pH.

The core biosynthetic pathway is relatively simple (Fig. 9), involving only a few enzymatic steps (Vogt, 2002; Christinet *et al.*, 2004; Sasaki *et al.*, 2005; Hatlestad *et al.*, 2012; Polturak *et al.*, 2016; Sunnadeniya *et al.*, 2016; Timoneda *et al.*, 2019). l-DOPA is a key compound in the pathway, needed for the formation of both BA and *cyclo*-DOPA. In some plants, l-DOPA also condenses with BA to form the betaxanthin dopaxanthin. l-DOPA is derived from tyrosine through hydroxylation by CYP450 l-DOPA OXIDASE enzymes belonging to the CYP76AD clade (CYP76AD1/5/6/15). l-DOPA is converted to BA through an extradiol cleavage performed by l-DOPA 4,5-DIOXYGENASE (DODA). For betacyanin production, a CYP76AD enzyme (CYP76AD1/3) closely related to the l-DOPA OXIDASE is involved. It performs both the l-DOPA-forming reaction and the oxidation that converts l-DOPA to *cyclo*-DOPA. This dual functionality allows betacyanin and betaxanthin biosynthesis to be uncoupled so that either or both types of betalain can be produced in the same cell.

The biosynthetic enzymes are thought to have arisen from duplication of genes involved in other pathways and subsequently neofunctionalized for betalain biosynthesis. *DODA* is part of the LigB gene family, which is present in all land plant lineages. Phylogenetic, sequence and functional analyses suggest that an additional clade of LigB genes arose early in the evolution of the Caryophyllales order, and *DODA* evolved from within this additional clade (Christinet *et al.*, 2004; Brockington *et al.*, 2015; Chung *et al.*, 2015; Bean *et al.*, 2018). Some species are thought to have subsequently lost betalain biosynthesis and regained anthocyanin production; these have lost *DODA* genes (Brockington *et al.*, 2015; Timoneda *et al.*, 2019). Phylogenetic analysis indicates that the *CYP76AD* lineage underwent two gene duplication events within Caryophyllales, giving rise to three paralogous lineages that include the genes required for betalain biosynthesis (Brockington *et al.*, 2015; Timoneda *et al.*, 2019). Functional and phylogenetic data combined suggest that tyrosine hydroxylation alone was the ancestral function of the CYP76AD in the Caryophyllales lineage, and the ability to convert l-DOPA to *cyclo*-DOPA evolved later (Sunnadeniya *et al.*, 2016). An additional deregulated version of AROGENATE DEHYDROGENASE that facilitates increased accumulation of the tyrosine precursor is thought to have arisen prior to the evolution of specific betalain biosynthetic enzymes (Lopez-Nieves *et al.*, 2018; Timoneda *et al.*, 2019). Some glycosylation enzymes are able to work on both flavonoid and betalain substrates, so the betalain enzymes were probably ‘co-opted’ from the flavonoid pathway (Vogt, 2002; Sasaki *et al.*, 2005; Polturak *et al.*, 2016; Timoneda *et al.*, 2019).

Betalain production evolved in plant taxa that originally had anthocyanin production, and it is thought that betalain biosynthesis may have arisen multiple times (Brockington *et al.*, 2011; Timoneda *et al.*, 2019). The manner in which betalains arose to replace anthocyanins and the evolutionary drivers for it are unresolved, although a potential evolutionary path has been suggested recently (Timoneda *et al.*, 2019). Betalain and anthocyanin production are mutually exclusive in extant taxa, although transgenics that can produce both types of pigment demonstrate that production of both together is not damaging to the cell (Polturak *et al.*, 2017). Whether anthocyanin biosynthesis was lost before or after betalain biosynthesis arose, or whether there was a switch from one pathway to the other, is not known. As the *R2R3MYB* transcription factor genes that activate betalain biosynthesis are similar to those that regulate anthocyanin biosynthesis (Hatlestad *et al.*, 2015), a mechanism is available for switching off one pathway and simultaneously activating the other. Betalainic species do retain some parts of the flavonoid pathway – such as for flavonol and proanthocyanidin biosynthesis. Thus, the genetic blocks preventing anthocyanin production are loss of function of a specific anthocyanin biosynthetic step and/or lack of transcriptional activation of the required genes.

## ENVIRONMENTAL TRIGGERS AND PATTERNS OF RED PIGMENTATION

When considered from a general perspective, the environmental triggers for induction of red vegetative pigmentation are remarkably consistent among terrestrial algae and the many different groups of land plants. If species show stress-induced red pigmentation, then strong PAR in conjunction with a secondary stressor, such as cold, drought or nutrient imbalance, is commonly one of the triggering factors. In angiosperms, excess PAR, cold, drought, nutrient imbalance and pathogen or herbivore attack are all known inducing factors for both anthocyanin and betacyanin production. Indeed, the general patterns of production of anthocyanins and betacyanin are very similar with regard to both tissue localization and triggers for induction (Davies, 2015). This may, in part, reflect the co-option of the R2R3MYB transcription factor that originally regulated anthocyanin production to the regulation of betalain biosynthesis (Hatlestad *et al.*, 2015). Fronds of the fern *Azolla* turn from green to red because of the production of large amounts of 3-deoxyanthocyanin in response to strong sunlight and temperature fluctuation (Fig. 6), as well as during interaction with microbial species (Cohen *et al.*, 2002*b*). Similarly, many mosses, including *Ceratodon purpureus* and varied *Sphagnum* species, become intensely red pigmented by sphagnorubin when experiencing abiotic stress from excess PAR, dehydration, nutrient imbalance, temperature below 5 °C or pathogen attack (Gerdol *et al.*, 1998; Bonnett *et al.*, 2010; Manninen *et al.*, 2011). Liverwort species may produce red pigmentation (presumably auronidin) in response to excess PAR, nutrient imbalance or during interaction with symbiotic or pathogenic microbes (Hooijmaijers and Gould, 2007; Humphreys *et al.*, 2010; Albert *et al.*, 2018; Kubo *et al.*, 2018; Carella *et al.*, 2019; Davies *et al.*, 2020). Moreover, within terrestrial algae, excess PAR can induce the production of varied phenolic red pigments in a range of Zygnematophyceae species, and red carotenoids in some Chlorophyceae species. Under saturating PAR conditions, phenolic pigments of glacier-living algae are produced as the predominant light-absorbing compounds, at >10-fold the concentrations of chlorophyll *a* (Williamson *et al.*, 2020). Thus, despite the type of red pigmentation differing greatly in both structure (e.g. anthocyanins, auronidins, sphagnorubins and carotenoids) and cellular localization (vacuole, cell wall, lipid bodies or external mucilage), the biosynthetic pathways are responding to the same general stressors.

It is much more common for flavones or flavonols rather than anthocyanins to be induced by UVB (Ferreyra *et al.*, 2021). Simple anthocyanin-glycosides do absorb in the UVB region of the spectrum (Fig. 7), but with lower molar absorption coefficients than flavone- or flavonol-glycosides. Aromatic acylation of anthocyanins significantly adds to the ability to absorb UVB radiation (Giusti *et al.*, 1999). However, most of the >300 different acylated anthocyanins reported are from floral tissues, and they have not been reported as UVB tolerance compounds in leaves or vegetative tissue (Andersen and Jordheim, 2006). The liverwort *Cephaloziella varians* becomes intensely red pigmented, presumably from auronidin, in response to UVB radiation in Antarctic conditions (Newsham *et al.*, 2005). This is in contrast to the UVB induction of flavones in *M. polymorpha* (Clayton *et al.*, 2018) or the common presence of cell wall-located colourless UVB-absorbing compounds in bryophytes (Clarke and Robinson, 2008; Soriano *et al.*, 2018*a*, *b*). Overall, induction of colourless UVB-absorbing flavonoids or other compounds rather than red pigments seems to predominate as a response to UVB exposure.

As with many other aspects of pigmentation, data on environmental triggers for production, *in planta* localization and the specific pigment structures produced are abundant for angiosperms but lacking for other land plant lineages. For example, mosses are known to be able to produce both 3-deoxyanthocyanins and sphagnorubins, yet there is little knowledge about the signals activating the different biosynthetic pathways, or indeed if both, one or neither pathway is present in different branches of the moss phylogenetic tree.

Although general patterns and triggers of induction may be shared, when pigmentation is compared at a species level many differences emerge. Depending on the angiosperm species examined, anthocyanins may be prevalent in emerging foliage, senescing foliage, in areas of pest damage, following pathogen attack, in stems, in trichomes or in roots, tubers or other below-ground organs. Even when produced in the same tissues at similar times, the localization within tissues can vary, such as being only in the adaxial epidermis, being limited to sub-epidermal mesophyll cells or being only in the abaxial epidermis. Anthocyanins are also abundantly produced in trichomes (Jordheim *et al.*, 2016) and stems (Cooney *et al.*, 2015) of some species. Studies on environmental triggers for, and tissue localization of, betacyanin production are fewer than for anthocyanins. However, as mentioned earlier, the studies suggest that betacyanins have similar tissue distributions and inductive signals to those reported for anthocyanins (Lee and Collins, 2001; Mosco, 2012; Chung *et al.*, 2015). This includes production in subterranean organs of some species, such as the tubers of the food crops beet (*Beta vulgaris*) and ulluco (*Ullucus tuberosus*), and in trichomes (including pigmented bladder cells that accumulate sodium and chloride ions in high concentrations; Dassanayake and Larkin, 2017). However, as with anthocyanins, varied triggers are reported depending on the species, including light quantity and quality, UVB radiation, elevated salinity (200 mmol L^− 1^), ROS and low temperature (below 10 °C), nitrogen or phosphorus deficiency, metal ion stress and plant senescence (Ibdah *et al.*, 2002; Wang and Liu, 2007; Wang *et al.*, 2007; Hayakawa and Agarie, 2010; Jain and Gould, 2015*a*, *b*; Jain *et al.*, 2015; Klein *et al.*, 2018; Parida *et al.*, 2018; Henry *et al.*, 2019; Izzo *et al.*, 2019; reviewed in Davies *et al.*, 2018; G. Li *et al.*, 2019; Tossi *et al.*, 2021). Although there are no published studies for stress functions in relation to autumnal senescence, abundant red pigmentation from betacyanins is a widely reported autumnal phenomenon of *Salicornia* species in saline environments (Cárdenas-Pérez *et al.*, 2021). Betaxanthins often have different accumulation patterns from those of betacyanins. However, they may be produced in the same cells and can alter the observed colour (Chung *et al.*, 2015). When comparing the pigments of vascular and non-tracheophyte plants, there is a marked difference in sub-cellular localization: anthocyanins and betacyanins are almost always located in the vacuoles while auronidins and sphagnorubins are cell wall bound.

With such varied patterns of production, it can be difficult to draw general conclusions that relate pigment localization to function. For anthocyanins, this has contributed to many alternative proposals about function that have made it almost impossible to find a unified explanation for their evolution. Nevertheless, whether located in the mesophyll (Hughes and Smith, 2007; Hughes *et al.*, 2014) or adaxial epidermis (Hughes *et al.*, 2007; Landi *et al.*, 2014), or present in the abaxial epidermis of understorey plants in shade conditions (Hughes *et al.*, 2014), trichomes (Jordheim *et al.*, 2016) or stems (Cooney *et al.*, 2015), anthocyanins can provide effective photoprotective screens. This supports a photomodulation function. However, some localizations suggest that certain pigments have other primary functions, which can be illustrated by two examples. In *Marchantia* species, auronidins are commonly produced in the ventral scales on the underside of the plant that provide a barrier between the meristem and soil (Fig. 5; Davies *et al.*, 2020). This is not suited to protecting against damaging PAR, but does fit an anti-pathogen function. In some halophytes, betalains are induced in the bladder cells, which facilitate salt secretion (Dassanayake and Larkin, 2017). Notably, red carotenoids are not reported as stress-induced pigments in land plants, despite the presence of the biosynthetic pathways and their demonstrated benefits as PAR stress-related compounds in algae. This indicates some advantage offered by production of red phenolic pigments beyond direct PAR screening, even if they vary in structural aspects. The varied production and sub-cellular localizations of the different phenolic pigments may reflect specific functions delivered by the individual pigment structures in addition to screening PAR, and we examine this in the final section of this review.

## HOW DOES PIGMENT STRUCTURE RELATE TO FUNCTION?

Anthocyanin pigments are based on the flavylium cation that allows more flexibility in structure than other non-charged chemical species and, since structure relates directly to function, there are numerous benefits in having a system that can adapt to changing environmental stimuli. Nature uses the versatility of the positively charged anthocyanin core structure to give rise to an abundance of colours through various multi-state chemical species (Fig. 8). The thermodynamic and kinetic properties of the flavylium multi-states are dependent on the localized pH as well as substituent effects of functional groups that decorate the core structure (Pina *et al.*, 2021). In addition to pH, light and temperature can modulate a range of reactions mediated by acid–base equilibria, hydration (particularly at the C2 position), tautomerism and isomerization (*cis*–*trans*) reactions. As such, these compounds can vary from colourless to yellows, reds, purples and blues. A similar effect has been observed in the positively charged auronidins that give rise to colours ranging from yellow to red to purple (Alejo-Armijo *et al.*, 2020). However, at physiological pHs, the colour range is likely to be more restricted. Unlike the anthocyanins, the auronidins have no equivalent C2 position available for hydration, so their structures are less flexible. Furthermore, the auronidins are stabilized *in vivo* through a close association with the cell wall, the mechanics of which are yet to be fully resolved.

Since flavylium ions are susceptible to degradation, there are a range of reported mechanisms that plants use to stabilize or change pigment colour, including acylation (particularly with aromatic precursors) and hydrogen bonding (enhanced through glycosylation); ionic interactions (e.g. metal ions); and hydrophobic (e.g. in molecular stacking) effects. These changes in both electron density (due to substituent effects) and electrostatic interactions also affect the hue of colours observed. Furthermore, through co-pigmentation, the colour of anthocyanins can be further intensified and their structures stabilized through interactions with other molecules (Pina *et al.*, 2021). Anthocyanins are relatively planar in shape and can form intramolecular stacks with similarly shaped molecules, including simple phenolics when co-localized. Stacking and other physical measures can lead to hyperchromic (increase in absorbance) and/or bathochromic (increase in wavelength) shifts in absorbance. As mentioned earlier, metal ion addition generally leads to bluer hues while complex interactions involving co-pigmentation lead to the supramolecular structures found in some blue flowers, e.g. cornflowers (Houghton *et al.*, 2021). Undoubtedly some of these intense colours have developed to attract pollinators as part of an ever-evolving chemical ecology, but whether there are further roles beyond photoprotection has yet to be determined.

Information is comparatively sparse for other red pigments. Betalains maintain more consistent spectral absorbance over a greater pH range than anthocyanins (Stintzing and Carle, 2004), and, as mentioned earlier, 3-deoxyanthocyanidins are more stable against hydrophilic attack. Whether the higher stability of these pigments is important for their functions *in planta* has not been explored. However, it is interesting that CAM photosynthesis, which is prevalent in betalainic species, can involve marked changes in cellular pH, with the vacuolar sap of these assimilatory cells in CAM reaching pH 4 or lower (Winter and Smith, 2022). Betalains could have an advantage over anthocyanins for pigmentation at these acidic pHs. Anthocyanins would require extensive co-pigmentation or other cellular interactions for stability at these pHs, but that would not be required for betalains (for which co-pigmentation has not been reported). Betalains also have higher extinction coefficients than anthocyanins, meaning less pigment is required to generate the same depth of colour, and betaxanthins autofluoresce (Gandía-Herrero *et al.*, 2005). As with many other aspects of non-anthocyanin pigments, the significance of these pigment characteristics has not been explored, although it is suggested that fluorescence is unlikely to be a signal in biocommunication (Iriel and Lagoria, 2010). Similarly, there are no data to confirm whether the structures of auronidins and sphagnorubins offer advantages for binding in the cell wall or forming phenolic polymers. Auronidin–riccionidin dimers have been isolated (termed riccionidin B) (Kunz *et al.*, 1993), providing a basis for formation of a polymer that could provide a physical barrier against pathogens, replicating one of the functions of lignin in vascular plants. Auronidin in the cell walls of *M. polymorpha* does indeed reduce penetration by hyphae of *Phytophthora palmivora* (Carella *et al.*, 2019). Antioxidant functions are often proposed as central to anthocyanin stress tolerance properties. However, the anthocyanins considered in these studies are water-soluble, vacuolar-located glycosides, while auronidins and sphagnorubins are generally cell wall-bound aglycones, again suggesting differing cellular functions.

## IS THERE A COMMON CORE FUNCTION OF RED PIGMENTS?

The hypothesis that anthocyanins protect leaves of plants facing biotic or abiotic stressors dates back to Pringsheim (1879), and is now a widely accepted function for foliar anthocyanins (Landi *et al.*, 2015, 2021; Gould *et al.*, 2018; Renner and Zohner, 2019, 2020). A direct PAR screening function of red pigments may seem self-evident – after all, colourless compounds are common to the UVB stress response while red pigments that can absorb PAR wavelengths are induced by excessive visible light exposure. However, whether this is the only cellular mechanism(s) underlying the mitigating effects of red pigments, or indeed their most important, is yet to be elucidated. Most debate arises from two properties of anthocyanins (and most red pigments) that contribute to their ability to reduce both the incidence and the severity of photo-oxidative damage and may account for their capacity to assist with a plethora of environmental stress: (1) intercepting a portion of supernumerary photons that would otherwise strike the chloroplasts (Feild *et al.*, 2001), the so-called sunscreen prerogative; and (2) quenching stress-triggered ROS, thereby acting as powerful antioxidants. There is an analogous debate on how phenolics provide UVB tolerance. Flavones, flavonols and hydroxycinnamic acids all absorb in the UVB section of the light spectrum, allowing for a direct screening function to protect systems such as PSII (Booij-James *et al.*, 2000; Takahashi and Badger, 2011). However, it has been argued that the antioxidant function of flavonoids is of more importance in reduction of UVB-related damage (Agati *et al.*, 2020; Landi *et al.*, 2015; Ferreyra *et al.*, 2021). Measuring screening or ROS-scavenging functions separately *in planta* is challenging, as reduction of PAR or UVB radiation by direct screening may reduce production of ROS. Moreover, various additional functions not relating to these two cellular properties are also proposed for anthocyanins and add to the debate – including leaf warming, microbial defence, anti-feedants and, notably for current functional theories, communication with the biotic environment.

A heated debate has occurred in the last few decades concerning the functions of anthocyanins in senescing autumn leaves, in which the ‘co-evolution’ hypothesis has been proposed as an alternative to the photoprotection hypothesis, and the arguments put forward in the debate are useful for examining general functional theories for red pigments. Briefly, while the photoprotection hypothesis considers anthocyanins as capable of screening excessive light and/or preventing formation of and scavenging stress-triggered ROS (Landi *et al.*, 2015; Gould *et al.*, 2018), the co-evolution hypothesis suggests that anthocyanins in senescing leaves provide an honest warning signal of defence against insects that migrate to trees, generating preference for green leaves with lower contents of defensive chemicals (Archetti *et al.*, 2009). Lately, new data have renewed the debate (Renner and Zohner, 2019; Pena-Novas and Archetti, 2020,2021*a*; Agati *et al.*, 2021; Hughes *et al.*, 2022; Joly, 2022). A comprehensive analysis by Renner and Zohner (2019) of 711 deciduous woody species confirmed a primary photoprotective function for anthocyanins and reported that cyanic leaves occur more frequently in species inhabiting areas characterized by lower temperatures and higher solar irradiance during autumn/winter, and hence suffering more severe ‘light stress’ during leaf senescence. In response, Pena-Novas and Archetti (2020) argued against Renner and Zohner’s results and in favour of the co-evolution hypothesis. However, we consider that the arguments made against photoprotective functions raise outdated misconceptions of anthocyanins that are worth addressing point by point. We examine the specific arguments in the context of both the co-evolution hypothesis and the relative importance of sunscreening or antioxidant action in contributing to photoprotection.

### Anthocyanins are not optimally located for photoprotection in leaf tissues

The core argument here is that the vacuolar localization of anthocyanins reduces their usefulness for acting on chloroplast-generated ROS (Karageorgou and Manetas, 2006). However, the short-lived ROS generated by chloroplasts are rapidly converted into H_2_O_2_. H_2_O_2_ can quickly pass lipid bilayers such as the tonoplast, and the tonoplast is generally adjacent to chloroplasts as the vacuole accounts for >90 % of photosynthetic cells’ total volume (Yamasaki *et al.*, 1997). Indeed, anthocyanin-rich cells accumulate less H_2_O_2_ than their acyanic counterparts (Gould *et al.*, 2002; Zhang *et al.*, 2012). In addition, Zipor and Oren-Shamir (2013) demonstrated that vacuolar anthocyanins are effective electron donors for vacuolar peroxidases, thereby suggesting a further mechanism for reducing ROS. Anthocyanins are frequently sub-epidermal in foliar tissues, while the concentration in epidermal cells might be suggested as optimal for light screening. However, even if sub-epidermal anthocyanins are principally antioxidants, they still screen some potentially damaging light.

### Anthocyanins mainly absorb green light that is relatively harmless to chloroplasts

This is an old misconception (Manetas, 2006) that needs to be overcome. It is true that anthocyanins mainly absorb in the yellow–green portion of the PAR spectrum that is relatively poorly absorbed by chlorophylls (Fig. 7), although the carotenoids that assist chlorophylls in the light-harvesting process do extend the absorbance of PAR wavebands somewhat into the green region. However, green light is essential for plants under shade (Smith *et al.*, 2017), as occurs for most anthocyanin-rich understorey species. Green light also interacts with photoreceptors (Landi *et al.*, 2020) and is essential for photosynthesis of the deeper mesophyll layers (Liu *et al.*, 2021), which are reached by red/blue-depleted light because of the strong absorbance of upper layers of the mesophyll. It has also been recently demonstrated that absorbance of blue light by anthocyanins has to be seriously considered (Landi *et al.*, 2021), especially when anthocyanins are synthesized in their acylated forms under stress conditions (Jordheim *et al.*, 2016). Finally, green light is used more efficiently than red or blue light at high photosynthetic photon flux densities (Landi *et al.*, 2020), and supplementation with green light does cause higher photoinhibition (Hughes *et al.*, 2005).

### Photoprotection should translate to higher nutrient resorption in senescing leaves, and this does not consistently occur in red-leafed tree species

Observations that anthocyanins facilitate nutrient recovery during autumn (Hoch *et al.*, 2003) led to a general assumption that, if the photoprotection hypothesis is correct, higher nutrient resorption in reddening compared with yellowing senescing leaves should be consistently observed (Pena-Novas and Archetti, 2021*b*), thereby conferring an ecological advantage from having anthocyanin-rich leaves. While it is true that resorbed nutrients are directly available for plant growth but nutrients recycled through litterfall may not become available for plant uptake for a long time (Aerts, 1996), nutrient resorption consequences of photoprotection need to be considered in the context of the specific ecosystem. Indeed, it is an open question whether nutrient-deficient species that evolved in low-nutrient availability environments should still exhibit greater resorption efficiency when not growing in their natural, low-fertile habitat (Hughes *et al.*, 2022). Therefore, nitrogen resorption studies conducted with individuals out of their natural habitat (Pena-Novas and Archetti, 2021*b*) could generate misleading data. Additionally, nitrogen resorption analyses are usually conducted at ‘leaf level’, but a more robust approach would include an integrated overview at ‘whole-canopy level’, given that the progressive loss of foliage by plants could lead to changes in nitrogen recycling and a re-organization of morpho-anatomical and leaf photosynthetic traits (Escudero and Mediavilla, 2003). For example, the efficiency of nitrogen recovery before leaf fall in different woody species can be dependent on: duration of the abscission period (Del Arco *et al.*, 1991); leaf life span and related different nitrogen recycling abilities (Pornon and Lamaze, 2007), especially when including evergreen species in the comparison (Pena-Novas and Archetti 2021*b*; Hughes *et al.*, 2022); and leaf mass changes upon senescence (L. Li *et al.*, 2019). Additionally, alternative analyses of the same current datasets have produced contrasting findings against (Pena-Novas and Archetti, 2021*b*) or in favour (Joly, 2022) of the photoprotection hypothesis. Future studies on nitrogen resorption capacity in red vs. yellow senescing leaves should therefore take a more comprehensive approach that includes consideration of the many influencing factors. It is also notable that autumn climatic conditions can generate red pigmentation of photosynthetic tissues that are not shed, such as in moss and fern species, arguing against nutrient reallocation – and co-evolution – hypotheses in such cases.

### Several reports have failed to demonstrate photoprotection by anthocyanins

One of the initially more challenging arguments against the photoprotection hypothesis is the failure in some studies to find any advantage of red over green/yellow leaves for photoinhibition amelioration in plants under stress. However, the definition of the term ‘photoinhibition’ is not universally accepted. For example, in *sensu lato* it is described by ecophysiologists as the light-induced depression in the CO_2_ assimilation rate with a consequent reduction in the maximum quantum yields for CO_2_ uptake, a decrease in the convexity of the photosynthetic light response curve and, with prolonged exposure to excessive light, a decreased rate of light-saturated photosynthesis (Long *et al.*, 1994). Nevertheless, in most studies on the photoprotective role of anthocyanins, the possible reduction/prevention of photoinhibition was simply estimated as the preservation of the PSII photochemical efficiency, either maximal (*F*_v_/*F*_m_) or operational (Φ_PSII_). This is too simplistic an approach, given that: PSII is only part of the photosynthetic apparatus and effects of other components such as PSI and/or Calvin–Benson cycle are not measured; photochemistry of PSII is only part of the dissipation mechanisms used for energy quenching; and chlorophyll fluorescence analyses provide an estimation of the leaf photosynthetic potential based on the analysis of just a shallow layer of chloroplasts over the leaf lamina. Therefore, it is not surprising that although many studies find that anthocyanins correlate with reduction of photoinhibition (i.e. higher *F*_v_/*F*_m_), others fail to uncover any photoprotective benefits (for a critical review, see Gould *et al.*, 2018). It is conceivable that contemporary re-examination of older contradictory cases could find opposite results (some examples are reported in Hughes *et al.*, 2022). Most reported studies were conducted in controlled conditions with low photon flux density (Gould *et al.*, 2018). However, the term ‘photoprotection’ only has a real meaning in saturating light conditions, whether this be relatively high PAR or lower PAR amounts in conditions of compromised photosynthesis (such as extreme temperatures). In addition, the presence of anthocyanins implicates a suite of morpho-anatomical and biochemical changes which strongly affect the interception, entry and management of light by the leaf (Hughes, 2011). Finally, red vs. green leaf performance may differ during the day (Tattini *et al.*, 2017).

In summary, only broader analyses of the photosynthetic process at the whole-leaf level (e.g. through gas exchange parameters and the real CO_2_ conductance to mesophyll in green compared with red individuals), as well as measurements of oxidative stress markers, can unequivocally provide information on the contribution to photoprotection of light screening by anthocyanins relative to antioxidant roles. This latter point has been controversial for decades (Neill and Gould, 2003) and, as mentioned earlier, is also debated for colourless flavonoids in terms of UVB radiation screening vs. antioxidant function. However, nowadays both the anthocyanin light screening and the antioxidant prerogative have started to be considered as two faces of the same coin (Landi *et al.*, 2015). To further complicate the picture, anthocyanins are usually produced in young or senescent leaves, which are more vulnerable to environmental stress as they have reduced photosynthetic capacity, yet in some ecological studies these leaves are compared with mature green leaves regardless of the strong differences in photosynthetic apparatus (Manetas *et al.*, 2002). Many of these considerations for examining the benefits of anthocyanins for photoprotection in the context of the alternative co-evolution hypothesis also apply to the question of why plants produce biosynthetically costly pigments when there is already an effective NPQ antioxidant system: it is necessary to accurately replicate the stress conditions experienced by anthocyanic tissues in nature and make appropriate experimental measurements. The use of genetic lines with constitutive red or green phenotypes and/or alterations to alternative stress tolerance mechanisms, preferably near-isogenic mutants or transgenics, would be a great advantage for such comparisons.

## FUNCTIONS BEYOND PHOTOPROTECTION

The question we address in the final part of this review regards what additional functions red pigments may have beyond photoprotection (i.e. considered as light sunscreen and/or antioxidant function), and what this may indicate about evolution of the biosynthetic pathways. In relation to photosynthetic function, one possibility is that red pigments provide benefit as ‘carbon sinks’ to help avoid transitory feedback regulation of photosynthesis (Adams *et al.*, 2013; Lo Piccolo *et al.*, 2020). Indeed, there is an indissoluble link between sugar signalling and anthocyanin induction (Solfanelli *et al.*, 2006), and anthocyanin biosynthesis can represent a valid carbon sink able to maintain the activity of the Calvin–Benson cycle, thereby avoiding energy (ATP) and reducing power (NADPH) accumulation in photosystems that would otherwise generate ROS. Indeed, the fact that photoinhibited leaves typically contain an overabundance of non-structural carbohydrates strongly suggests that photoinhibition of photosynthesis in those leaves might be reduced by the biosynthesis of carbon-based secondary metabolites, thereby possibly reducing the sugar accumulation and the feedback regulation of photosynthesis (Lo Piccolo *et al.*, 2020). The carbon-buffering role exerted by anthocyanins can also represent a mechanism to balance the C/N ratio in senescing leaves, thereby extending the leaf life span of red- vs. green-leafed tree species (Lo Piccolo *et al.*, 2018; Vangelisti *et al.*, 2020). Therefore, it is conceivable that the carbon-buffering role should be included as another mechanism through which anthocyanins confer photoprotection, besides their antioxidant and screening capabilities (Gould *et al.*, 2018; Lo Piccolo *et al.*, 2018, 2020). This mechanism could also explain the accumulation of anthocyanins in abaxial leaf epidermises (which are not an optimal location for screening PAR) in species living in the understorey; for these shade-adapted species, transitory sunflecks can exert a severe photoinhibitory effect (Hughes *et al.*, 2014). The carbon-buffering hypothesis also matches the recent nutrient-centred theory of anthocyanin function proposed by Hughes *et al.* (2022). The authors hypothesized that the evolution of species with red leaves in autumn was driven by soil fertility, with leaf reddening a response to low quantities of foliar nutrients.

For all the aforementioned reasons, the term ‘photomodulation’ rather than the classical ‘photoprotection’ seems to be more apt for future use, and better describes the role of anthocyanins as mediators in light-driven reactions. Whether this is the case for other red pigments has yet to be determined, since there have been few studies of this nature. For many of the pigments, an initial examination supports a core photoprotective function centred on direct screening, rather than antioxidant or secondary photomodulation functions. The cell wall-bound, probably insoluble, accumulation of auronidins and sphagnorubins and external accumulation in mucilage of some algal pigments argues against an antioxidant function. While betalain production has many similar stress triggers to those of anthocyanin production, since betalains contain nitrogen they are not suited to modulating the C/N balance and would not be expected to show the same responses to sugar status.

Understanding the physiological roles of the non-anthocyanin red pigments is important for proposing the possible evolutionary drivers of pigmentation in general, and research on non-anthocyanin model species is needed. The evidence reviewed here indicates that a core photoprotection function by direct screening is a major driver that has given rise to auronidin, sphagnorubin, 3-deoxyanthocyanin, anthocyanin, betalain and various algal red pigments that can all absorb in the visible wavelengths, i.e. generating a red pigment is the key evolutionary driver. That different lineages have evolved to produce different pigments could reflect the chances of mutation. However, as betalains are thought to have replaced anthocyanins on more than one occasion (Brockington *et al.*, 2011), that at least suggests a functional evolutionary driver. We propose that each of the red pigment structures shares the core screening function, but they have additional functions for interactions with the abiotic and biotic environment. While it is possible these differing functionalities were factors influencing the initial evolution of the biosynthetic pathways, including co-evolution with other organisms (Rausher, 2001), it is perhaps more probable that once red pigment production had evolved for photoprotection, the pathway was adapted for secondary functions that conferred an evolutionary advantage. For anthocyanins, the diversification of angiosperms and anthocyanin secondary modifications, and the versatility of the core anthocyanin structure, have generated a myriad of proposed additional functionalities that have contributed to the challenge of finding a unified explanation for anthocyanin production. Some of these are well supported as replacing the photoprotection role of anthocyanins, such as blue flower colouration for pollination. For others, notably the co-evolution hypothesis for autumn leaf colour, it is necessary to find solid supporting evidence before they can replace the photoprotection hypothesis. For auronidins and 3-deoxyanthocyanidins, there is strong evidence of anti-pathogen or anti-herbivore functions. For betalains, evidence is growing of specific activities in salinity tolerance.

If red pigmentation has arisen many times, then what might the pigment status of the last common ancestor of land plants have been? The lack of anthocyanins in hornworts and liverworts, in the context of the proposed monophyly of bryophytes, makes it difficult to reach a conclusion. It is possible that the 3-deoxyanthocyanin pathway was present in the last common ancestor but lost in hornworts, liverworts and some mosses, with ‘replacement’ by auronidins or sphagnorubins. More whole-genome sequences from non-angiosperm species would greatly help in addressing this question.
